# The Protective Effects of HJB-1, a Derivative of 17-Hydroxy-Jolkinolide B, on LPS-Induced Acute Distress Respiratory Syndrome Mice

**DOI:** 10.3390/molecules21010077

**Published:** 2016-01-11

**Authors:** Xiaohan Xu, Ning Liu, Yu-Xin Zhang, Jinjin Cao, Donglin Wu, Qisheng Peng, Hong-Bing Wang, Wan-Chun Sun

**Affiliations:** 1 Central Laboratory, The Second Clinical Hospital, Jilin University, Changchun 130041, China; xuxiaohan2015@outlook.com (X.X.); liu_ning@jlu.edu.cn (N.L.); jinjincao90@163.com (J.C.); 2 Key Laboratory of Zoonosis, Ministry of Education, Institute of Zoonosis, Jilin University, Changchun 130062, China; qishengpeng@yahoo.com; 3 School of Life Sciences and Technology, Tongji University, Shanghai 200092, China; 4 Key Laboratory of Molecular Enzymology & Engineering, Ministry of Education, College of Life Science, Jilin University, Changchun 130012, China; chongfengxing@163.com; 5 Jilin Provincial Center for Disease Control and Prevention, Changchun 130062, China; dl_wu@163.com

**Keywords:** Acute respiratory distress syndrome (ARDS), NF-κB, MAPK, 17-hydroxy-jolkinolide B, edema

## Abstract

Acute respiratory distress syndrome (ARDS),which is inflammatory disorder of the lung, which is caused by pneumonia, aspiration of gastric contents, trauma and sepsis, results in widespread lung inflammation and increased pulmonary vascular permeability. Its pathogenesis is complicated and the mortality is high. Thus, there is a tremendous need for new therapies. We have reported that HJB-1, a 17-hydroxy-jolkinolide B derivative, exhibited strong anti-inflammatory effects *in vitro*. In this study, we investigated its impacts on LPS-induced ARDS mice. We found that HJB-1 significantly alleviated LPS-induced pulmonary histological alterations, inflammatory cells infiltration, lung edema, as well as the generation of inflammatory cytokines TNF-α, IL-1β and IL-6 in BALF. In addition, HJB-1 markedly suppressed LPS-induced IκB-α degradation, nuclear accumulation of NF-κB p65 subunit and MAPK phosphorylation. These results suggested that HJB-1 improved LPS-induced ARDS by suppressing LPS-induced NF-κB and MAPK activation.

## 1. Introduction

Acute respiratory distress syndrome (ARDS) is a devastating disease, which is characterized by a severe, acute lung inflammatory responses, then damaging the alveolar-capillary barrier diffusely, flooding protein-rich edema fluid into airspaces, resulting in severe gas-exchange abnormalities [1,2]. ARDS is associated with several clinical disorders, including sepsis, pneumonia, aspiration of gastric contents, and major trauma [3]. Among these, sepsis is the most common cause of ARDS in human [2,4]. Currently, major therapeutic strategies of ARDS are still supportive care measures, such as protective ventilation and supportive fluid conservative [5], no pharmacological treatments have yet proven effective at reducing mortality. Thus, it is critical to explore the innovative therapies and effective medications for ARDS.

LPS, a major pro-inflammatory component of Gram-negative bacteria cell walls, initiates robust inflammatory response via binding to toll like receptor 4 (TLR4) and activating TLR4-MyD88 dependent signaling pathway. Animals exposed to LPS suffer from ARDS by increasing inflammatory and chemotactic cytokine production and subsequent inflammatory cell sequestration in lung tissues [6]. A larger number of photochemical components have been reported to have protective role in LPS-induced lung injury or mortality via suppressing NF-κB activation and MAPK phosphorylation [7,8,9,10].

The ent-abietane diterpenoid compounds, isolated from the dried root of *Euphorbia fischeriana*, including jolkinolide B (JB), 17-hydroxy-jolkinolide B (HJB) and 17-hydroxy-jolkinolide A (HJA) have been reported to show anti-inflammatory effects [9,11,12]. In our previous study, we found HJB-1, a HJB derivative, possesses much more effective anti-inflammatory activity than HJB [12]. However, the *in vivo* anti-inflammatory effect of HJB-1 remains unclear.

The present study aimed to investigate the protective effect of HJB-1 on LPS-induced ARDS mouse model and further clarify the mechanism by which HJB-1 mitigate LPS-induced ARDS.

## 2. Results

### 2.1. Effects of HJB-1 on LPS-Induced Lung Histopathologic Changes

**Figure 1:**
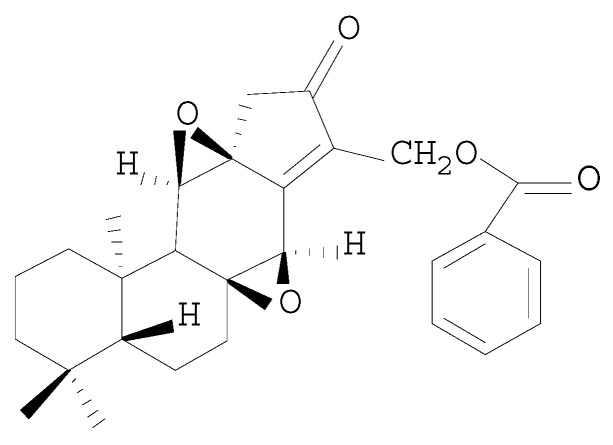
Chemical structure of HJB-1.

**Figure 2:**
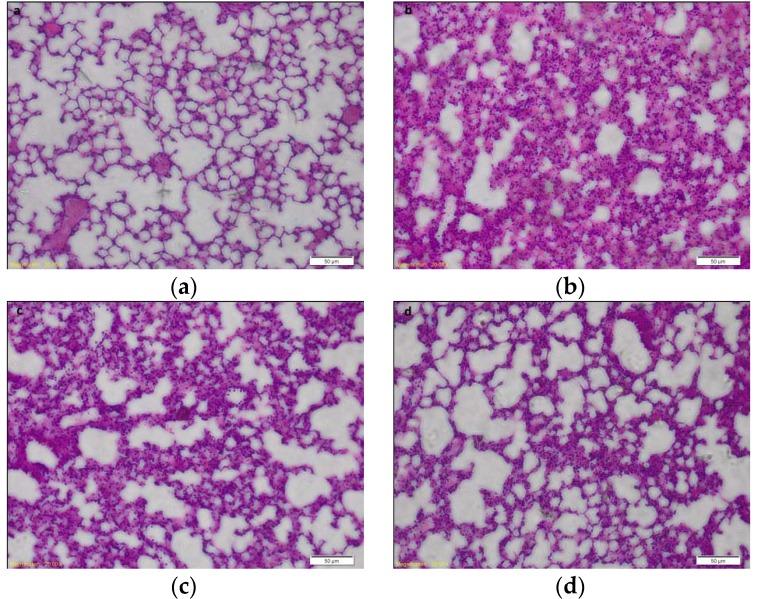

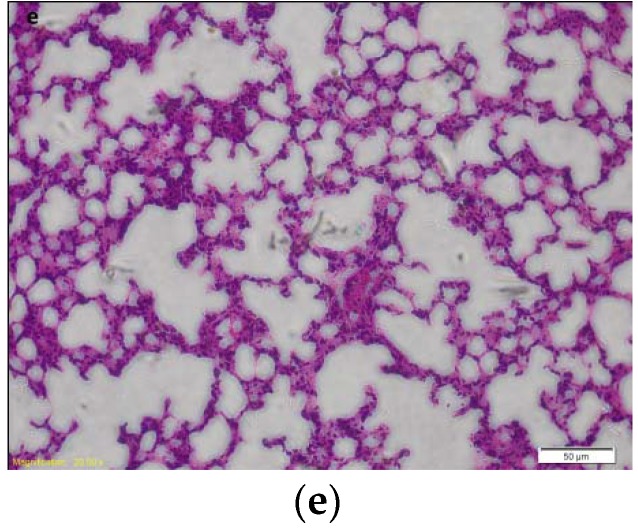
The effects of HJB-1 on the histopathological change in the lungs of LPS-induced ARDS mice. Representative sections of lung tissues from various experimental groups were stained with hematoxylin and eosin. HJB-1 (2 and 10 mg/kg, i.p.) or Dex (5 mg/kg, i.p.) was given 1 h before LPS treatment. Lungs (*n* = 6) from each experimental group were processed for histological evaluation at 24 h after LPS treatment. (**a**) Control group (saline treatment); (**b**) LPS group; (**c**): HJB-1 (10 mg/kg) + LPS group; (**d**) HJB-1 (2 mg/kg) + LPS group and (**e**) Dex (5 mg/kg) + LPS group (magnification 200×).

Previous study indicated that HJB-1 exhibited stronger anti-inflammatory effects on LPS-stimulated peritoneal macrophages than HJB. The chemical structure of HJB-1 was shown in Figure 1. To evaluate the effects of HJB-1 on LPS-mediated ARDS in mice, we examined the histological changes of lungs in different groups. As shown in Figure 2b, lung sections obtained from LPS group exhibited significant pathologic changes, including inflammatory cell infiltration, pulmonary congestion and alveolar wall thickening. However, pretreatment with HJB-1 (2 and 10 mg/kg) significantly attenuate these pathological changes in dose-dependent manner (Figure 2c,d).

### 2.2. HJB-1 Attenuated LPS-Induced Pulmonary Vascular Permeability and Lung Edema

**Figure 3:**
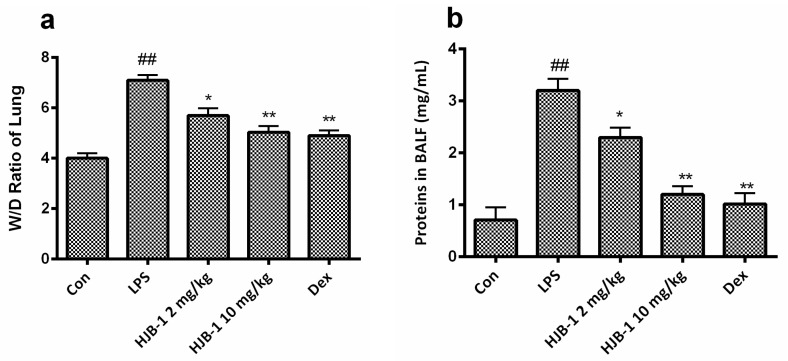
The effects of HJB-1 on LPS-induced pulmonary vascular permeability and lung edema. HJB-1 (2 and 10 mg/kg, i.p.) or Dex (5 mg/kg, i.p.) was given 1 h before LPS treatment. (**a**) Lung edema was determined by measuring wet/dry ratio of lung tissues in LPS-induced ARDS mice; (**b**) Pulmonary vascular permeability was determined by measuring the protein levels of BALFs in LPS-induced ARDS mice. Each value is presented as the mean ± SEM (*n* = 6). ## *p* < 0.01 *vs.* control group; * *p* < 0.05 and ** *p* < 0.01 *vs.* LPS group.

LPS challenge results in an increasing pulmonary capillary leakage, which leads to lung edema in LPS-induced ARDS mice. As shown in Figure 3, the lung W/D ratio and total protein levels in the BALF were significantly increased after LPS challenge(*p* < 0.01), revealing the formation of lung edema. However, pretreatment with HJB-1 (2 and 10 mg/kg) apparently attenuated the level of lung W/D and total protein levels in the BALF compared with that in LPS group (*p* < 0.05). These results suggested that HJB-1 could dose dependently inhibit lung edema in LPS-induced ARDS mice.

### 2.3. HJB-1 Reduced Total Cells Number in the BALF of LPS-Induced ARDS Mice

**Figure 4:**
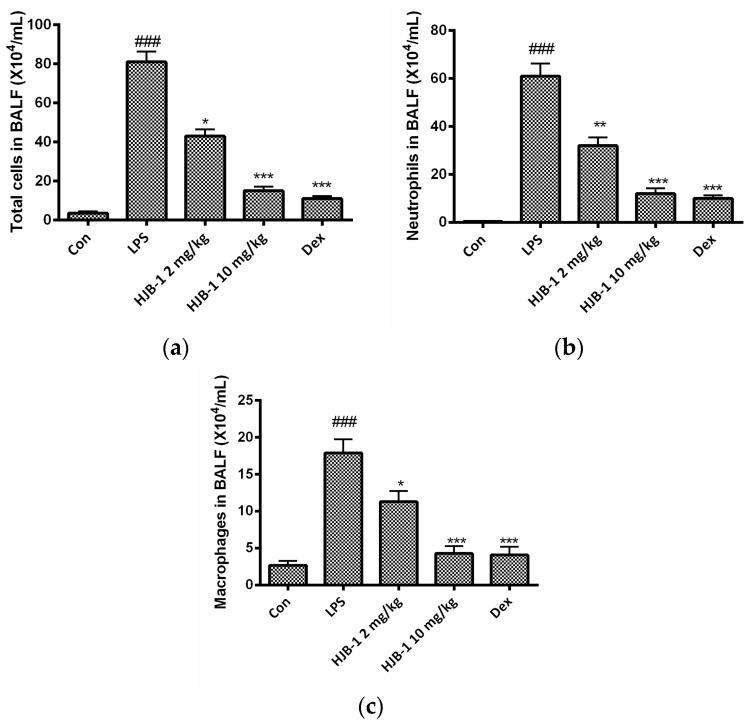
The effects of HJB-1 on inflammatory cell numbers in the BALF of LPS-induced ARDS mice. HJB-1 (2 and 10 mg/kg, i.p.) or Dex (5 mg/kg, i.p.) was given 1 h before LPS treatment. BALF was collected 24 h after LPS treatment and (**a**) total cell numbers; (**b**) neutrophils; and (**c**) macrophages in BALF were counted. Each value is presented as the mean ± SEM (*n* = 6). ### *p* < 0.001 *vs.* control group; * *p* < 0.05, ** *p* < 0.01 and *** *p* < 0.001 *vs.* LPS group.

To determine the effects of HJB-1 on the migration and infiltration of pulmonary cells, the total cell numbers, neutrophils and macrophages in the BALF of LPS-induced ARDS mice were counted 24 h after LPS administration. As shown in Figure 4, there was a significant increase in the number of total cells, neutrophils and macrophages in the BALF of the mice after being exposed to LPS (*p* < 0.001). However, pretreatment with HJB-1 (2 and 10 mg/kg) dose-dependently reduced the number of total cells, neutrophils and macrophages compared to LPS-induced ARDS mice (*p* < 0.05).

### 2.4. HJB-1 Alleviated the MPO Activity of Lung Tissues in LPS-Induced ARDS Mice

**Figure 5:**
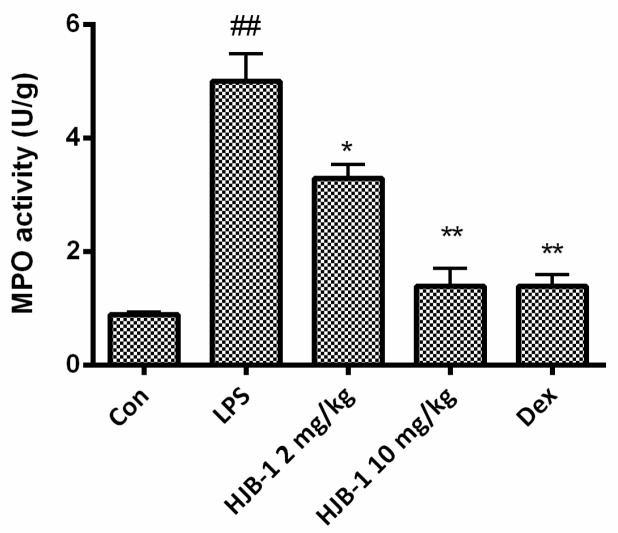
HJB-1 attenuated MPO activity of lung tissues in LPS-induced ARDS mice. HJB-1 (2 and 10 mg/kg, i.p.) or Dex (5 mg/kg, i.p.) was given 1 h before LPS treatment. Lung tissues was collected 24 h after LPS treatment and the MPO activity of collected lung tissues were examined. Each value is presented as the mean ± SEM (*n* = 6). ## *p* < 0.01 *vs.* control group; * *p* < 0.05 and ** *p* < 0.01 *vs.* LPS group.

The MPO activity of lung tissues was examined to evaluate the neutrophil accumulation within pulmonary tissues of LPS-induced ARDS mice. As shown in Figure 5, MPO activity is higher in the LPS group compared with the control group (*p* < 0.01). Administration of HJB-1 (2 and 10 mg/kg) caused a significant reduction in MPO activity in a dose-dependent manner (*p* < 0.05). These results demonstrated that HJB-1 suppressed excessive infiltration of neutrophils into the lung tissues in LPS-induced ARDS mice.

### 2.5. Effects of HJB-1 on Inflammatory Cytokines Production in BALF of LPS-Induced ARDS Mice

**Figure 6:**
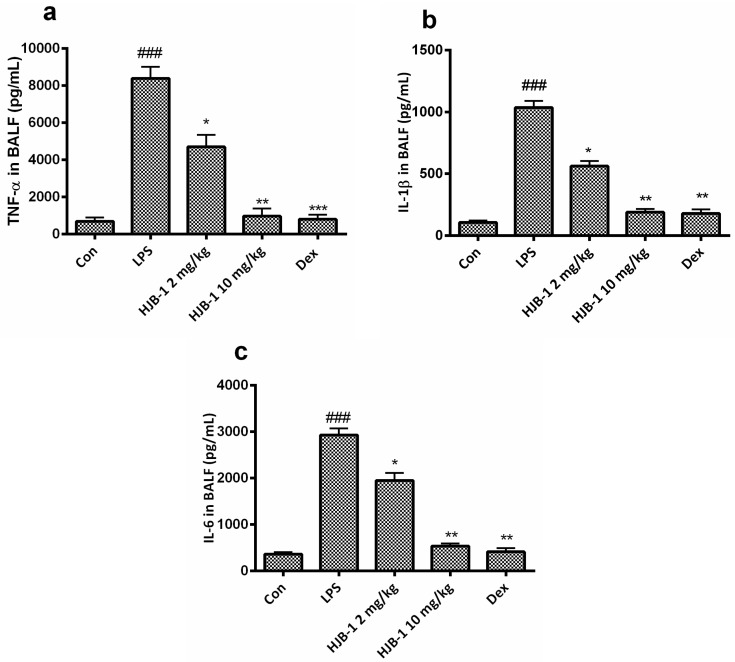
HJB-1 decreased inflammatory cytokine levels in BALF of LPS-induced ARDS mice. HJB-1 (2 and 10 mg/kg, i.p.) or Dex (5 mg/kg, i.p.) was given 1h before LPS treatment. BALF was collected 24 h after LPS treatment and inflammatory cytokines in BALF, including (**a**) TNF-α; (**b**) IL-1β; (**c**) IL-6 were measured by ELISA. Each value is presented as the mean ± SEM (*n* = 6). ### *p* < 0.001 *vs.* control group; * *p* < 0.05, ** *p* < 0.01 and *** *p* < 0.001 *vs.* LPS group.

To investigate whether HJB-1 improved LPS-induced ARDS via its anti-inflammatory activity, Inflammatory cytokines, including TNF-α, IL-1β and IL-6 in BALF of LPS-induced ARDS mice were examined by ELISA. As shown in Figure 6, LPS challenge apparently increased the production of TNF-α, IL-1β and IL-6 in BALF (*p* < 0.001). However, pretreatment with HJB-1 (2 and 10 mg/kg) significantly reduced the generation of TNF-α, IL-1β and IL-6 in BALF of LPS-induced ARDS mice in a dose-dependent manner (*p* < 0.05).

### 2.6. Effects of HJB-1 on NF-κB Activation in LPS-Induced ARDS Mice

**Figure 7:**
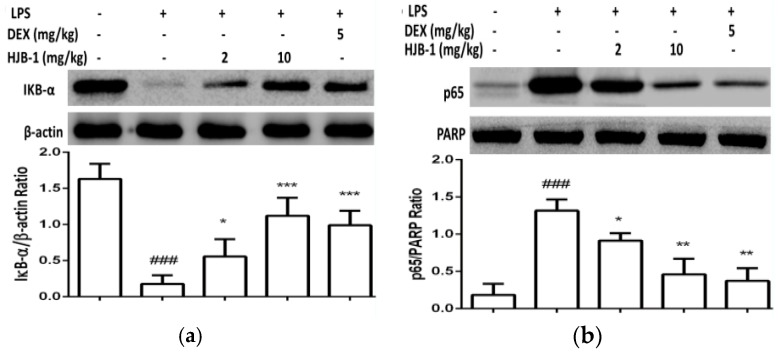
The effects of HJB-1 pretreatment on NF-κB activation in the lung tissues of LPS-induced ARDS mice. HJB-1 (2 and 10 mg/kg, i.p.) or Dex (5 mg/kg, i.p.) was given 1h before LPS treatment. Lung tissues were collected 24 h after LPS treatment and (**a**) IκB-α degradation; (**b**) NF-κB p65 subunit in nuclear extracts were examined by Western blot. The relative quantificationof target proteins was calculated by comparison of the band density levels between samples. The values shown in the graphs represent the mean ± SEM (*n* = 6). ### *p* < 0.001 *vs.* control group; * *p* < 0.05, ** *p* < 0.01 and *** *p* < 0.001 *vs.* LPS group.

To investigate whether HJB-1 inhibits inflammatory cytokines induction in LPS-induced ARDS mice through blocking NF-κB signaling, we examined the effects of HJB-1 pretreatment on NF-κB activation in the lung tissues of LPS-induced ARDS mice. As shown in Figure 7, LPS challenge significantly increased the degradation of IκB-α and nuclear accumulation of NF-κB p65 subunit (*p* < 0.001). However, pretreatment with HJB-1 (10 and 2 mg/kg) dose-dependently inhibited degradation of IκB-α and nuclear accumulation of NF-κB p65 subunit (*p* < 0.05). These results suggested that HJB-1 pretreatment suppressed NF-κB activation in LPS-induced ARDS mice.

### 2.7. Effects of HJB-1 on MAPK Phosphorylation in LPS-Induced ARDS Mice

**Figure 8:**
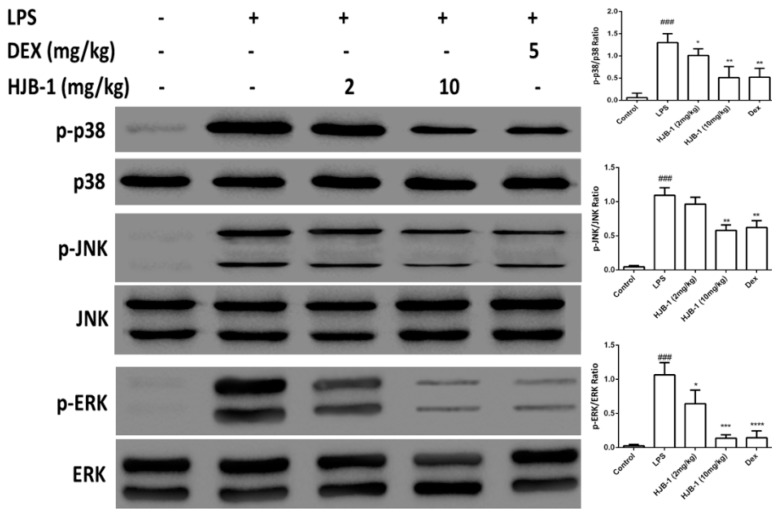
The effects of HJB-1 on MAPKs phosphorylation in the lung tissues of LPS-induced ARDS mice. HJB-1 (2 and 10 mg/kg, i.p.) or Dex (5 mg/kg, i.p.) was given 1 h before LPS treatment. Lung tissues were collected 24 h after LPS treatment and the phosphorylation of MAPKs, including p38, JNK and ERK, was examined by Western blot. The relative quantificationof target proteins was calculated by comparison of the band density levels between samples. The values shown in the graphs represent the mean ± SEM (*n* = 6). ### *p* < 0.001 *vs.* cntrol group; * *p* < 0.05, ** *p* < 0.01 and *** *p* < 0.001 *vs.* LPS group.

As shown in Figure 8, LPS challenge significantly increased the phosphorylation levels of MAPKs including p38, ERK and JNK compared to the control group (*p* < 0.001). However, pretreatment with HJB-1 (2 and 10 mg/kg) significantly suppressedLPS-induced phosphorylation of p38, ERK and JNK (*p* < 0.05). These results indicated that HJB-1 dose-dependently suppressed the activation of MAPK signaling in LPS-induced ARDS.

## 3. Discussion

In our previous study, we reported that HJB-1, a HJB derivative obtained via modifying the 17-hydroxy group of HJB, exhibited stronger anti-inflammatory activity than HJB [12]. Moreover, the *in vivo* effects of HJB-1 on inflammatory disorders including ARDS/ALI remain unclear. In the present study, we investigated the protective effects of HJB-1 on LPS-induced ARDS mice. Our results demonstrated that HJB-1 alleviated LPS-induced lung edema and pulmonary histological alterations. The generation of inflammatory cytokines TNF-α, IL-1β and IL-6 in BALF of LPS-induced ARDS mice were also decreased by pretreatment with HJB-1. The anti-inflammatory mechanism of HJB-1 is by suppressing LPS-induced NF-κB and MAPK activation.

Pulmonary edema is a representative symptom of ARDS [13]. The loss of alveolar-capillary barrier integrity is the prerequisite for the development of protein-rich pulmonary edema during ARDS [14]. The magnitude of pulmonary edema was evaluated by examining the lung W/D ratio. The results indicated that pretreatment with HJB-1 significantly alleviated the lung edema from the reduced W/D ratio and total protein concentration in BALF compared with LPS group. These results demonstrated that HJB-1 could suppress the filtration of protein-rich edema into the interstitial and alveolar spaces. The infiltration of inflammatory cells such as neutrophils into the lungs plays a crucial role in the process of ARDS by releasing several inflammatory mediators [15]. Pulmonary MPO activity is the indicator of neutrophil accumulation [16,17]. In this study, the results revealed that pretreatment with HJB-1 apparently reduced LPS-induced increases in MPO activity in the lung tissues. In addition, the numbers of total cell in BALF of LPS-induced ARDS mice were markedly decreased. These results suggested that pretreatment with HJB-1 significantly suppressed the infiltration of inflammatory cells into pulmonary alveolar space. Consistently, the histological observation also showed that HJB-1 improved LPS-induced pathologic changes, including inflammatory cell infiltration, pulmonary congestion and alveolar wall thickening.

Accumulating evidences indicated that inflammatory cytokines such as TNF-α, IL-1β and IL-6 play important roles in the pathogenesis of ARDS. Increased levels of TNF-α, IL-1β and IL-6 in the BALF have been noted in LPS-induced ARDS model [18,19,20,21]. These cytokines could amplify the inflammatory cascade and lead to the inflammatory injury. In the present study, HJB-1 markedly suppressed the generation of TNF-α, IL-1β and IL-6 in BALF of LPS-induced ARDS mice. These results suggested that the protective effects of HJB-1 on ARDS might be attributed to its suppressive effect on LPS-mediated inflammatory response.

NF-κB is a key transcriptional factor, which plays a pivotal role in the regulation of various inflammatory cytokines induced by external stimulation, such as LPS [12]. In quiescent cells, NF-κB is sequestered in the cytosol by its inhibitor IκB-α. On activation, NF-κB is released from the cytoplamic NF-κB- IκB-α complex and free NF-κB (a heterodimer of p65 and p50) is translocated into the nucleus, triggering the transcription of specific target genes, such as TNF-α, IL-1β and IL-6 [22]. MAPKs, including ERK1/2, p38 and JNK, also play a crucial role on inflammatory cytokine production induced by LPS. Once stimulated by LPS, MAPKs were phosphorylated and activated [23,24]. In the present study, we found that HJB-1 markedly suppressed LPS-induced IκB-α degradation, nuclear accumulation of NF-κB p65 subunit and MAPK phosphorylation. These results demonstrated that HJB-1 exerted its anti-inflammatory effect via preventing activation of NF-κB and MAPK.

## 4. Materials and Methods

### 4.1. Reagents

HJB-1 (purity ≥99%) was prepared by Tongji University as previously described [12]. Streptavidin HRP and anti-mouse IL-1β antibody pairs were purchased from Invitrogen (Carlsbad, CA, USA). Phosphatase inhibitor cocktail, *Escherichia coli lipopolysaccharide* (LPS, 055:B5) and protease inhibitor cocktail were purchased from Sigma (St. Louis, MO, USA). Anti-Mouse IL-6 polyclonal antibody, anti-Mouse TNF-α polyclonal antibody, biotin-conjugated polyclone mouse TNF-α antibody, biotin-conjugated polyclonal mouse IL-6 antibody and TMB substrate were from Ebioscience (San Diego, CA, USA). Antibodies against mouse p-JNK, p-p65, p-ERK1/2, p-p38, IκB-α, p38, JNK, p65, β-actin and ERK1/2 were purchased from Cell Signaling Technology (Beverly, MA, USA). All other chemicals used in the experiments were commercial products of reagent grade.

### 4.2. Animals

All mice were randomly divided into five groups (*n* = 12, each group): Control, LPS, LPS + HJB-1 (2 and 10 mg/kg) and LPS+DEX group. Half of each group was taken for the inflammation protein analysis, slicing and edema, and the rest used for BALF analysis. HJB-1 (2 and 10 mg/kg) and DEX (5 mg/kg) were administered intraperitoneally. Mice in control group and LPS group were given an equal amount of PBS. One hour later, mice were instilled intranasally (i.n.) 1 0μg of LPS in 50 μL PBS to induce lung injury. Control mice were given 50 μL PBS without LPS. 24 h after LPS instillation, animals were euthanized. The lung tissues and bronchoalveolar lavage fluid (BALF) were collected. All animal experiments were approved by “Institutional Animal Ethics Committee”, of Jilin university and performed in accordance with the Jilin university ethnic committee guideline for the Care and Use of Laboratory Animal (SCXK 2015-0003).

### 4.3. Histopathologic Evaluation of the Lung Tissue

For Histopathological assessment of lung injury, the lungs were harvested without lavage collection and fixed in 10% formaldehyde. After fixation, the lungs were embedded in paraffin, cut into 5 mm sections, and stained with hematoxylin and eosin (H & E). Pathological changes of lung tissues were observed under a light microscope.

### 4.4. Wet-to-Dry Lung Weight Ratio (W/D Ratio)

After the mice were euthanized, the lungs were immediately dissected and rapid weighted to record as the wet weight. Then the tissues were baked in an incubator at 80 °C for 48 h to obtain the dry weight. The ratio of wet lung weight to dry lung weight was calculated to assess the extent of lung edema.

### 4.5. BALF Collection and Cell Count

BALF was collected as described previously [25]. Briefly, after euthanasia, tracheostomy was performed and intubated with a tracheal cannula. BALF collection was performed by lavaging the airways and lungs three times with cold PBS in a total volume of 1.5 mL. The BALF samples were centrifuged at 700 *g* for 10 min at 4 °C. The cell pellets were resuspended in PBS (1 mL) and the total cell counts were determined using a hemocytometer in the BALF. Differential cell counts were performed on slides that were prepared by cytocentrifugation and Diff-Quick staining (Imeb, San Marcos, CA, USA). Total protein concentrations in the supernatants of BALF samples were examined using BCA assay kit (Thermo, Rockford, IL, USA) according to the manufacturer’s instructions to evaluate vascular permeability in the airways. Proteins were expressed in milligram protein per milliliter BALF.

### 4.6. MPO Activity Assay

Mice were euthanized 24 h after LPS challenge. Lung tissues were homogenized and fluidized in extraction buffer to obtain 5% of homogenates. Then the homogenates were centrifuged at 13,000 *g* for 30 min at 4 °C and used for MPO assay. MPO activity was measured using test kits purchased from Nanjing Jiancheng Bioengineering Institute (Nanjing, China) according to the manufacture’s instruction.

### 4.7. Measurement of Inflammatory Cytokines in BALF

Levels of the cytokines, including TNF-α, IL-1β and IL-6, in the supernatants of BALF samples, were measured by ELISA as previously described [9,12,26]

### 4.8. Western Blotting

Lung tissues were harvested at 24 h after LPS administration, then frozen immediately in liquid nitrogen for storage until homogenization. Proteins were extracted from the lungs using T-PER Tissue Protein Extraction Reagent Kit (Thermo) according to the manufacturer’s instructions, and the cytoplasmic and nuclear proteins were prepared using NE-PER Nuclear and Cytoplasmic Extraction Reagents (Thermo) according to manufacture’s instructions. The protein concentration was determined using BCA assay kit (Thermo). Equal amounts of protein (50 μg) were separated by SDS-PAGE and electro-transferred to a PVDF membrane (Millipore, Billerica, MA, USA). After being blocked for 1 h at room temperature with blocking buffer (3% BSA in TBST), the membrane was incubated overnight at 4 °C with the primary antibody and then incubated with an appropriate HRP-conjugated secondary antibody. Images were visualized and captured using MicrochemiChemiluminescence system 4.2 (DNR, Jerusalem, Israel).

### 4.9. Statistical Analysis

All of Data were expressed as means ± SEM. Statistically significant differences between groups were determined by ANOVA followed by *post hoc* Bonferroni test using Graphpad Prism 6 software. *p* < 0.05 was considered to be statistically significant.

## 5. Conclusions

In conclusion, the present study demonstrates the anti-inflammatory protective effect of HJB-1 on LPS-induced ARDS mice. The anti-inflammatory mechanism of HJB-1 may be associated with its suppression of NF-κB and MAPK activation, which consistent with our previous findings [12]. Therefore, HJB-1 may be a potential therapeutic agent for acute respiratory distress syndrome treatment.
